# Bibliography

**Published:** 1900-02

**Authors:** 


### Bibliography.

ANNUAL AND ANALYTICAL CYCLOPÆDIA OF PRACTICAL MEDICINE. By Charles E. de M. Sajous, M.D., and One Hundred Associate Editors, assisted by Corresponding Editors, Collaborators, and Correspondents. Illustrated with Chromo-Lithographs, Engravings, and Maps. Volume IV. The F. A. Davis Company, Publishers, Philadelphia, New York, and Chicago, 1899.

This really great Cyclopædia has reached its fourth volume, including, in its consideration of diseases, "Infants, Diarrhœal Diseases of," to "Mercury." These cover 622 pages.

The work has been fully noticed in previous issues of this journal, and any review of this fourth volume must be but a repetition of the favorable opinion entertained of it from the beginning. It is, in the opinion of the writer, one of the most valuable works of reference issued from the press. The editor says in his preface, "That it is with renewed pleasure that the editor places the fourth issue before his readers. The marked success implied has not only been due to the novel plan of the work,—a general article upon each disease, sustained by the salient points of the literature of the last ten years,—but also to the excellence of the general articles (presented in large type) written by the members of the associate staff. . ."

This volume contains, besides the article on "Insanity," a timely paper on the "Diarrhœal Diseases of Infants," by Professor Blackader, of Montreal; an elaborate paper on "Malarial Fevers," by Professor James C. Wilson and Dr. Thomas G. Ashton; articles on "Locomotor Ataxia," by Dr. W. B. Pritchard, of New York; on "Intubation," by Professor F. E. Waxham, of Chicago; "Diseases of the Liver," by Professor Alexander McPhedran, of Toronto; on "Meningitis," by Dr. Charles M. Hay, of Philadelphia. These are a few of the notable contributions. The editor has a valuable article on "Leprosy," largely based on personal observations of this disease.

The exhaustive article on "Diarrhœal Diseases of Infants" is a very valuable contribution to this much-written subject of infantile years. The causes and treatment of the various stages of this "symptom" of disease are very fully considered. The author defines diarrhœa "as a symptom only; a symptom indicative of increased motor activity and of increased and, perhaps perverted, secretory activity in the intestinal canal." Among the causes he enumerates "summer-heat directly prostrating the nervous system, over-excitement, and *occasionally the nerve irritation accompanying dentition.*" (Italics ours.)

The author gives statistics as to the age of children thus attacked, and these reveal "the fact that the great majority are under two years. . . . Holt has given us the statistics of 3000 cases of diarrhœa. . . . He finds that of the total number, infants under six months form fourteen per cent.; infants from six to twelve months, twenty-nine per cent.; infants from twelve to eighteen months, twenty-four per cent.; infants from eighteen to twenty-four months, seventeen per cent.; and children over two years, sixteen per cent."

While food and environments have largely to do with diarrhœal troubles in infants, and the author is justified in devoting a large portion of his article to these, yet, when he refers to dentition as an "occasional" cause, it is simply to express a profound ignorance of one of the principal, if not *the* principal, cause of diarrhœal disease at the ages named. The fact that infants, according to Holt's statistics, suffer at the rate of fourteen per cent. prior to six months, the period of eruption of the deciduous incisor teeth, and subsequently run up to twenty-nine per cent., with a gradual decrease until the close of the deciduous dentition, it would seem to indicate that the eruption of the series was a prominent factor in infantile disorders. It is, therefore, strange that in all the treatment given no allusion is made to the simple one adopted by every intelligent dental practitioner. If medical men would devote a portion of their work to the histological facts connected with both deciduous and permanent dentition, their articles would have a greater value in determining the origin of many of the so-called diseases of infantile years.

Space will not permit a review of the book as a whole. Each article is a treatise in itself, and is frequently made doubly valuable by the beautiful chromo-lithograph engravings and maps. The opinion previously expressed can only be repeated, that to all workers in general medicine, or the branches of the healing art, this series of volumes will be, when completed, the most valuable works of reference that have appeared for many years.

